# A long life in good health: subjective expectations regarding length and future health-related quality of life

**DOI:** 10.1007/s10198-015-0701-1

**Published:** 2015-06-16

**Authors:** David R. Rappange, Werner B. F. Brouwer, Job van Exel

**Affiliations:** Department of Health Policy and Management, Erasmus University Rotterdam, PO Box 1738, 3000 DR Rotterdam, The Netherlands

**Keywords:** Subjective expectations, Life expectancy, Quality of life, Health behaviour, Lifestyle, D84, H51, H75, I10

## Abstract

**Background:**

Subjective life expectancy is considered relevant in predicting mortality and future demand for health services as well as for explaining peoples’ decisions in several life domains, such as the perceived impact of health behaviour changes on future health outcomes. Such expectations and in particular subjective expectations regarding future health-related quality of life remain understudied. The purpose of this study was to investigate individuals’ subjective quality adjusted life years (QALYs) expectation from age 65 onwards in a representative sample of the Dutch generic public.

**Methods:**

A web-based questionnaire was administered to a sample of the adult population from the Netherlands. Information on subjective expectations regarding length and future health-related quality of life were combined into one single measure of subjective expected QALYs from age 65 onwards. This subjective QALY expectation was related to background, health and lifestyle variables. The implications of using different methods to construct our main outcome measure were addressed.

**Results:**

Mean subjective expected QALYs from age 65 onwards was 11 QALYs (range −9 to 40 QALYs). Individuals with unhealthier lifestyles, chronic diseases, severe disorders or lower age of death of next of kin reported lower QALY expectations. Indicators were varyingly associated with either subjective life expectancy or future health-related quality of life, or both.

**Conclusion:**

Extending the concept of subjective life expectancy by correcting for expected quality of life appears to generate important additional information contributing to our understanding of people’s perceptions regarding ageing and lifestyle choices.

## Introduction

The wish for a long and healthy life is often heard. Still, not everyone will live such a life. Differences in life expectancy and healthy life expectancy between groups remain large [1]. Many individuals will have subjective expectations regarding their own length of life and their future health-related quality of life, which may differ (substantially) from objective projections. Such subjective expectations remain understudied, especially regarding future health-related quality of life, but they may be relevant for a number of reasons.

First, subjective expectations regarding length and future health-related quality of life may be important if they influence decisions. If people have specific ideas about how old they will become and how they will become old, this may influence current decisions in several life domains. For instance, expectations may influence the decisions to invest in their future health and length of life or choices regarding pensions and savings. Therefore, understanding (the formation of) subjective expectations enables us to learn more about, and possibly influence, decision-making. For the health domain, this is important given the preventable mortality and morbidity attributable to modifiable, unhealthy health behaviours [2]. People who expect old age to be associated with low quality of life regardless of current investments may be less likely to engage in preventive actions. Moreover, individuals who expect ageing to be associated with unavoidable deterioration of health may also be less prone to use healthcare. For example, Sarkisian et al. [3] found that older adults with low expectations regarding ageing believed seeking healthcare to be less important for age-associated, modifiable ill-health conditions. As such, subjective expectations for length and future quality of life can influence current decisions. Especially when subjective expectations are inaccurate (for instance too pessimistic) this may result in non-optimal decisions.

Second, the demand for healthcare services and need for long-term care may increase as societies age and the proportion of elderly rises, which is the case in most developed countries (e.g., [4]). This poses important challenges for the future sustainability of healthcare systems and society in general, both in terms of financing and planning. Subjective expectations obtained from individuals, instead of actuarial data, may provide more insight into future healthcare needs and demands if they contain (private) information other than what is accounted for in actuarial data [5].

Third, subjective expectations regarding length and future health-related quality of life may also play a role in research. For instance, in explaining discount rates observed in experiments or when valuing health states using the time trade-off (TTO) method (see [6, 7]), these expectations may be important.

Several large household surveys include questions regarding longevity expectations, mostly elicited as subjective survival probabilities. Studies using these data have focused on the accuracy of such longevity expectations compared to actuarial figures (e.g., [8–12]) or investigated their ability to predict mortality (e.g., [10, 13–16]). Other research has studied these subjective survival probabilities in relation to (economic) decisions regarding retirement, saving and lifestyle (e.g., [9, 17–22]). In general, subjective survival probabilities contain information not found in objective measures, are found informative in predicting mortality, and are relevant for explaining economic and lifestyle decisions of individuals. Thus far, the study of subjective expectations regarding future health-related quality of life has received less attention. Recently, Péntek et al. [23] explored subjective expectations regarding future health and treatment effects among patients with rheumatoid arthritis (and their rheumatologists), and concluded that such expectations may be important in the context of treatment decisions and compliance. The authors advocated more work in this area.

Our study therefore set out to investigate these subjective expectations regarding length and also future health-related quality of life in more detail. It elaborates on previous work of Brouwer and Van Exel [24] and Péntek et al. [25] who studied (the accuracy of) expectations regarding both length and future health-related quality of life. Expectations regarding length of life were not based on survival probabilities in these studies, but directly elicited by asking respondents their expected age of death. Brouwer and Van Exel [24] found in a sample from the Dutch general public that individuals generally overestimate their life expectancy (males more than females), as had been found before [8], but (considerably) underestimate future quality of life from age 70 onwards. Furthermore, age, current health status and perception of own lifestyle compared to others each explained a significant part of the variance in the expectations regarding length and future quality of life. What is more, the average age of death of next of kin was related to subjective life expectancy. Péntek et al. [25] conducted a similar study in members of the general public in Hungary and found results which were largely in line with those from Brouwer and Van Exel [24].

In this paper, we present new data on subjective expectations regarding both length and future health-related quality of life. Our study adds to the previous two studies of Brouwer and Van Exel [24] and Péntek et al. [25] in a number of ways. First, Brouwer and Van Exel [24] combined two unrepresentative Dutch convenience samples from two independent studies, while Péntek et al. [25] used an unrepresentative Hungarian sample gathered through a Hungarian web journal. In our study, we used a representative sample of the Dutch general public instead. Second, in contrast to these two previous studies, we used a more elaborate set of background, health and lifestyle variables, which are potentially important in the context of subjective expectations. A final, specific feature of our study that adds to those reported by Brouwer and Van Exel [24] and Péntek et al. [25] is that we combine subjective expectations regarding length and future health-related quality of life into one single composite measure. In other words, we extend the concept of subjective life expectancy by adding (and correcting for) self-estimated quality of life during these years. Using this method, we assess the subjective expectations regarding the remaining number of life years after age 65 adjusted for the quality of life in these years lived. Moreover, we examine the relationship between these expectations and background characteristics, objective health indicators and, in particular, lifestyle, since subjective life expectancy is increasingly considered important in relation to lifestyle choices. We investigate whether this latter hypothesis holds for a measure that combines subjective life expectancy with expectations regarding future health-related quality of life. We also discuss the implications of using different methods to construct our composite expectations measure.

The remainder of this paper is structured as follows. First, we discuss our data, methods and analyses. In particular, we describe how we constructed our combined subjective measure of expectations. After that, we present our results. We end the paper with a discussion of our main results and the implications resulting from our findings.

## Materials and methods

### Data collection and outcome measures

For our study, we developed a web-based questionnaire that was administered to a sample of 18- to 65-year-olds from the Netherlands, representative in terms of age, gender and level of education. The overall objective of this survey was to investigate how Dutch people think about (future) health and choices in healthcare.

**Fig. 1:**
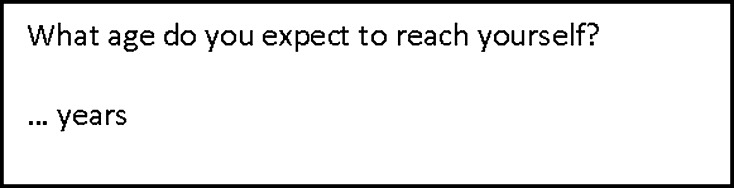
Question used for eliciting a point estimate of subjective life expectancy

We included a measure of subjective life expectancy as well as a measure of expected health-related quality of life in our survey to operationalize our main outcome variable ‘subjective future quality adjusted life years (QALYs) expectation from age 65 onwards’. The concept of (objective) QALYs is frequently applied in the evaluation and comparison of healthcare interventions [26], but not in the context of individuals’ subjective expectations.^1^ After introducing the concept of subjective expectations we elicited a point estimate of the subjective life expectancy for each respondent (see Fig. 1). Respondents were allowed to fill in any integer between 0 and 120. This method was successfully used before by Brouwer and Van Exel [24] and Péntek et al. [25].

**Fig. 2:**
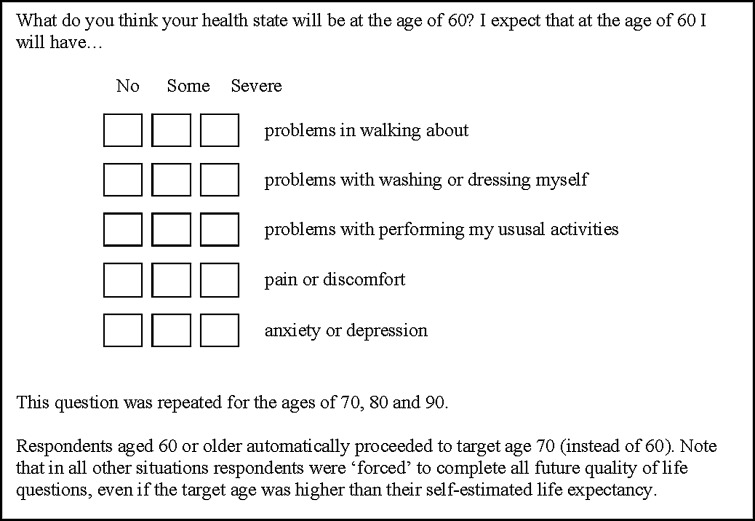
Question for eliciting expectations regarding future health, using the dimensions of the EuroQol-5D

Next, to elicit respondents’ current and expected future health states we employed the EQ-5D instrument ([27]; see also http://www.euroqol.org), as was done previously [24, 25]. The EQ-5D is a generic health-related quality of life instrument comprising five health dimensions: ‘mobility’, ‘self-care’, ‘usual activities’, ‘pain/discomfort’ and ‘anxiety/depression’. For each dimension the respondent could indicate to (expect to) experience ‘no problems’, ‘some problems’ and ‘extreme problems’. Thus, 243 distinct health states can be distinguished for which preference scores exist which were obtained from the general public [28]. The EQ-5D instrument was designed to measure current health. Figure 2 specifies how we asked questions regarding future health using the EQ-5D dimensions. This method was also used in the previous two studies [24, 25].

**Fig. 3:**
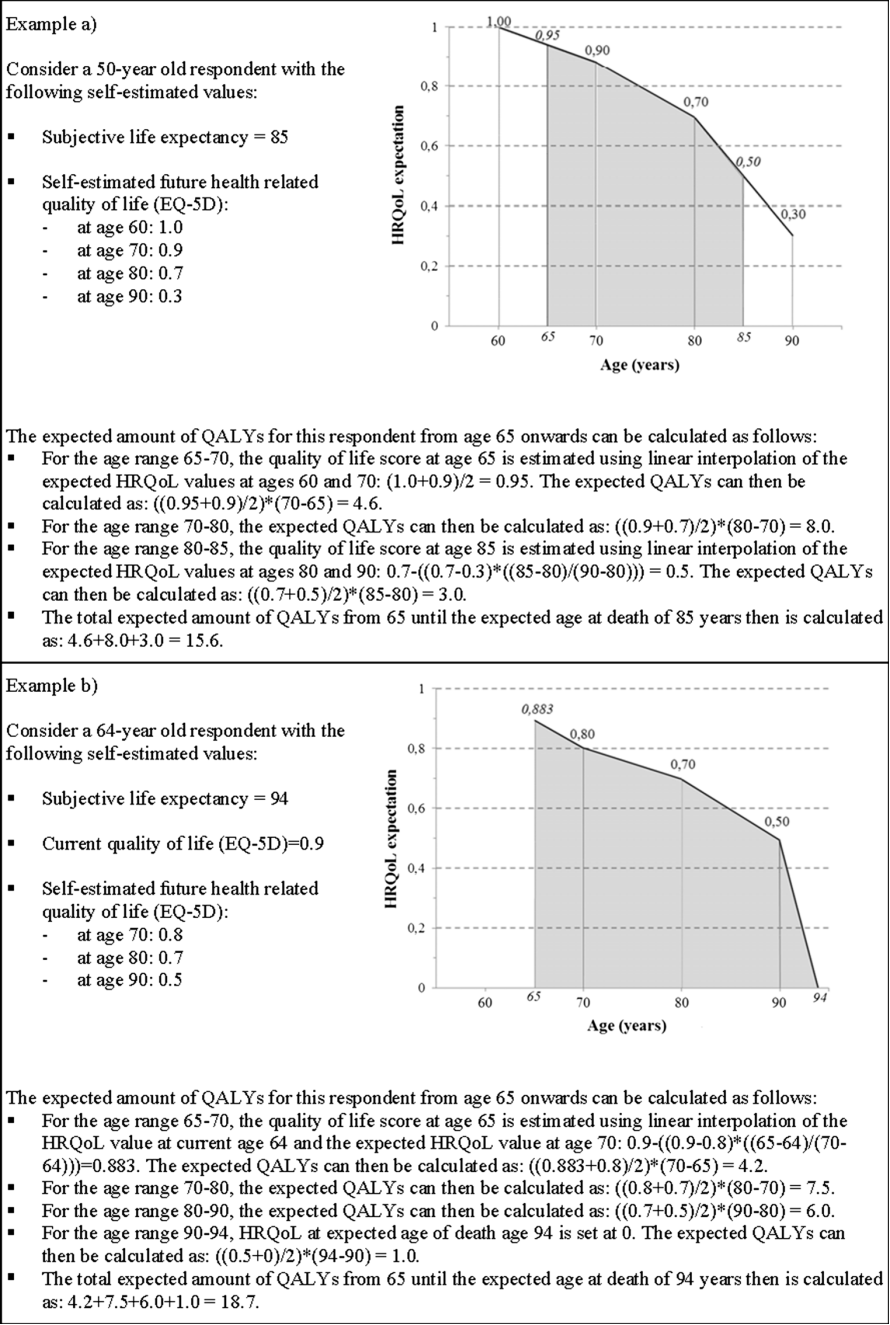
Computation method for combining expectations regarding length and future health-related quality of life into a single outcome variable measuring expectations regarding remaining QALYs from age 65 onwards

Combining the expectations regarding length and future health-related quality of life presented above provides us our main, single outcome variable, i.e., a measure of subjective expectations regarding the remaining amount of QALYs from 65 onwards. In Fig. 3 we present two examples to explain our computation method.

Since we had no information on respondents’ expected quality of life at time of death, except for those respondents that reported a subjective life expectancy equal to one of our target ages used for the quality of life questions, we imputed these scores. As can be seen in Fig. 3, we differentiated our imputation method according to the subjective life expectancy of respondents. For respondents who reported a subjective life expectancy of 90 or lower, we computed the quality of life at time of death based on the QALY scores of two subsequent target ages. Respondents with a subjective life expectancy higher than 90 were ascribed a quality of life score of 0 at time of death, since no information on quality of life expectations was available for ages higher than 90.

In order to retain all respondents while ensuring that the future QALY expectations for all respondents started at age 65, we imputed quality of life scores at age 65. For the respondents aged between 18 and 60 (60-year-olds not included), we used quality of life scores at age 60 and 70 to come up with a mean quality of life score at age 65 (see ‘example a’ in Fig. 3). For respondents aged between 60 and 65, we used their current self-reported health state and the expected quality of life score at age 70, as is the case in ‘example b’ from Fig. 3.

### Other variables/instruments

The survey included questions on socio-demographic characteristics, such as gender, age, marital status and net income. Moreover, respondents indicated their height and weight and were asked about the following lifestyle indicators: physical (in)activity, eating habits, smoking and alcohol consumption. Respondents were asked to indicate how many days a week they performed at least 30 min of (vigorous) exercise, such as walking, cycling or sports. The Dutch guidelines for healthy exercise require at least 30 min of exercise at least 5 times a week [29, 30]. Then, respondents reported how many days a week, on average, they ate healthily (i.e., balanced meals including a wide variety of food in the right proportions and amount). A minimum of 6 days per week was set to classify respondents as having a healthy diet. We distinguished non-smokers from occasional smokers and current smokers. Male and female respondents were considered heavy drinkers when their weekly amount of alcohol consumption exceeded 21 drinks and 14 drinks, respectively, or when consuming six drinks or more on one occasion at least once a week [31].

After general questions regarding the (past) presence of a severe disorder and any current chronic diseases (both physical and psychological), a vertical, visual analogue scale ranging from 0 (‘worst imaginable health’) to 100 (‘best imaginable health’) was used to obtain respondents’ own valuation of current health. A similar format was used to elicit a general happiness score. Respondents were also asked to state their preference between a shorter life in perfect health and a longer life in a less than perfect health state and to give an indication of the average age most of their next of kin had reached.

Finally, we used an instrument that measures expectations regarding ageing (ERA-12). This validated 12-item survey measures expectations regarding ageing on three domains of four items each, i.e. expectations regarding physical health, expectations regarding mental health and expectations regarding cognitive function. These three subscales combine to one general scale measuring expectations regarding ageing [3, 32].

### Descriptive statistics

First, sample characteristics are presented. Due to the way we constructed the survey, we avoided missing values on any of the variables. However, two respondents reported a bodyweight of 0 kg. We imputed these values based on height, gender and education in our sample.

We constructed a ‘lifestyle index’ based on the four aforementioned indicators of risky behaviour (based on Dutch health norms), i.e., smoking (on a daily basis), excessive alcohol consumption, physical inactivity and unhealthy diet. The index ranged from 0 to 4 with higher values indicating an unhealthier lifestyle. For example, a lifestyle index of 3 may indicate a person who smokes daily, drinks excessively and is physically inactive. For our analyses we combined groups 3 and 4 because of low numbers (2 %) in group 4.

Descriptive statistics of subjective expectations of life expectancy and future health-related quality of life expectations are presented. Subsequently, this is done for our main outcome variable, i.e. the subjective expectations of future QALYs from 65 onwards. Since the answers to the questions regarding subjective life expectancy and future health-related quality of life differed importantly (and therefore automatically also regarding our main outcome variable) between respondents from the age groups 18–59 and 60–65, we focused in particular on these differences throughout our analyses.

Finally, for validation purposes, we analysed the extent to which our measure of expectations regarding future QALYs remaining from age 65 onwards correlated with the 12-item ERA survey and its three 4-item subscales.

### Multivariate analysis

We used linear regression analysis to identify explanatory variables for the number of subjective expected QALYs from 65 onwards. Explanatory variables were included based on the previous findings of Brouwer and Van Exel [24] and Péntek et al. [25]. We defined four models, each model nested in the previous one, which successively introduced (1) socio-demographic characteristics and socioeconomic status, (2) health indicators, (3) age of death of next of kin and, finally, (4) the lifestyle index. Due to notably different results on our expectation variables for the groups 18–59 and 60–65, we included age both as a dummy variable, differentiating between both age groups, and as a continuous variable. Furthermore, we paid particular attention to the explanatory power of the lifestyle index/indicators and also conducted our regression analysis for men and women separately.

### Sensitivity analyses

We performed several sensitivity analyses in order to test several choices we made. Most importantly, an alternative computation method for the expected total amount of future QALYs from 65 onwards involves using a quality of life score of 0 at time of death for all respondents, instead of using the quality of life score at the subsequent target age. Alternatively, we altered our initial approach only for those respondents reporting a subjective life expectancy over 90 years old. Instead of assuming a quality of life score of 0 at time of death we used the reported quality of life score at target age 90 (i.e., assuming no decline from that point onwards). Other aspects that deserved attention regard (the elimination or adjustment of) possible outliers and the examination of the impact when age and lifestyle indicators are included differently into our regression analysis. We ran our multivariate analysis incorporating these adjustments. All analyses were conducted using STATA 11 IC (StataCorp, College Station, TX).

## Results

### Sample characteristics

A sample of 1223 respondents representative of the general population from the Netherlands in terms of age, gender and level of education completed the web-based survey. We excluded observations based on the time it took to complete the survey. In our sample, all respondents completed the survey between 5 and 62 min with mean length of almost 26 min (SD = 9.0 min). A small pilot exercise indicated that the minimal time necessary to complete the survey quickly but carefully was 15 min. Therefore, we excluded 157 respondents who completed the survey within 15 min (12.8 % of total sample). We also excluded respondents who reported a lower life expectancy than their age at the time of the interview (*n* = 3). Our final sample therefore consisted of 1064 respondents. The main sample characteristics are shown in Table 1.

**Table 1:** Sample characteristics, *n* = 1064

Variable	Category	%
Male (%)		50.1
Age [mean (SD)]	Range (18–65)	43.2 (13.6)
Educational level (%)^a^	Low	27.3
	Middle	42.0
	High	30.7
Marital status^b^	Living alone/divorced	32.2
	Married/living together	67.8
Have children (%)		60.2
(Self-) employed (%)		53.0
Income^c^	Low	30.1
	Middle	47.3
	High	22.7
Health (EQ-5D) [mean (SD)]	Range (−0.13:1)	0.84 (0.23)
Disorder (currently/ever) (%)		28.2
Chronic disease (%)		36.6
Health (VAS) [mean (SD)]	Range (0–100)	75.1 (16.5)
Happiness (VAS) [mean (SD)]	Range (0–100)	74.5 (18.0)
Obese (%)^d^		19.2
Physically active		50.9
Healthy diet (%)		47.5
Smoking (%)	Never	60.5
	Yes, sometimes	11.0
	Yes, daily	28.5
Alcohol consumption	No	35.9
	Moderate	52.6
	Excessive	11.5
Lifestyle index	0	20.5
	1	33.4
	2	32.4
	3 or 4	13.7
Next of kin’s age of death	<75	19.5
	75–85	53.7
	≥85	26.9

^a^Low: primary or lower secondary education; Middle: upper secondary education or post-secondary non-tertiary education; High: Bachelor, Master, Doctoral or equivalent

^b^The category ‘married/living together’ also included 37 respondents (3.5 %) who indicated ‘do not want to say/other’

^c^Low <1500; middle 1500–2999; high ≥3000 in euros

^d^‘Obese’ indicates BMI ≥30 kg/m^2^. Mean (SD) BMI = 26.4 (5.1)

Respondents excluded from the final sample were younger and more often male (*p* < 0.01), so that our final sample for analysis was no longer completely representative for the Dutch population aged 18–65 years old. Mean subjective life expectancy and subjective QALY expectation were not significantly different (*p* < 0.01) between included and excluded respondents.

### Subjective life expectancy

**Fig. 4:**
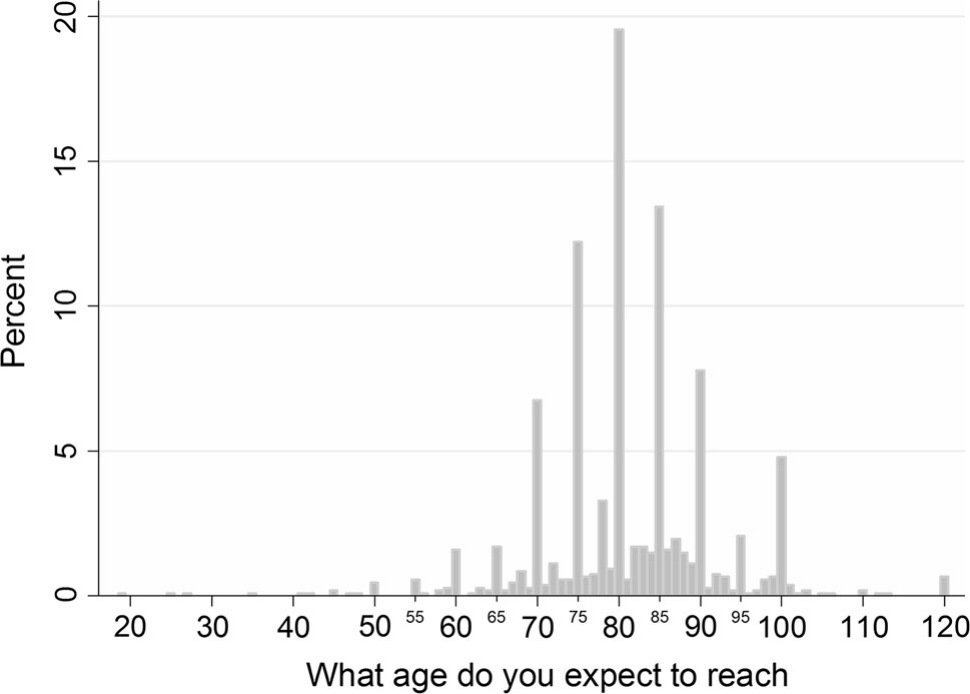
Distribution of subjective life expectancy (*n* = 1064)

The mean expected age of death in our sample was 81.1 years (SD = 10.9 years). Respondents reported life expectancies in a range between 19 and 120 years old. The distribution of these subjective life expectations is presented in Fig. 4. A considerable part of the respondents used round numbers in expressing their longevity expectation: 41.0 % of the predictions were rounded to tens (60, 70, 80, etc.) and 71.3 % to fives or tens (70, 75, 80, etc.). Clear peaks were present at 75, 80 and 85 (12.2, 19.5, 13.4 %, respectively). The time gap between the respondent’s age at the time of the survey and their subjective life expectancy ranged between 0 and 102 years and was on average 37.9 years (SD = 17.0). As expected, this time gap diminished as respondents’ age at the time of the interview increased. Analysis by age group showed that the mean subjective life expectancy was significantly higher in the group 60–65 compared to the group 18–59: 84.8 and 80.5 years, respectively [*t*(1062) = −4.4964, *p* < 0.001]. No variation in subjective life expectancy was found between respondents aged below 60.

### Subjective expectations regarding future health-related quality of life

Respondents were asked to report their expectations regarding future health-related quality of life at the target ages of 60 up to 90. Average scores declined steadily with age, from 0.77 to 0.69, 0.51 and 0.32 at the ages of 60, 70, 80 and 90, respectively [recall that respondents aged 60 or more (*n* = 143, 13.4 %) did not need to predict health at age 60]. The scores ranged from −0.329 to 1 at all ages, equalling the possible minimum and maximum scores according to the EuroQol system.

**Fig. 5:**
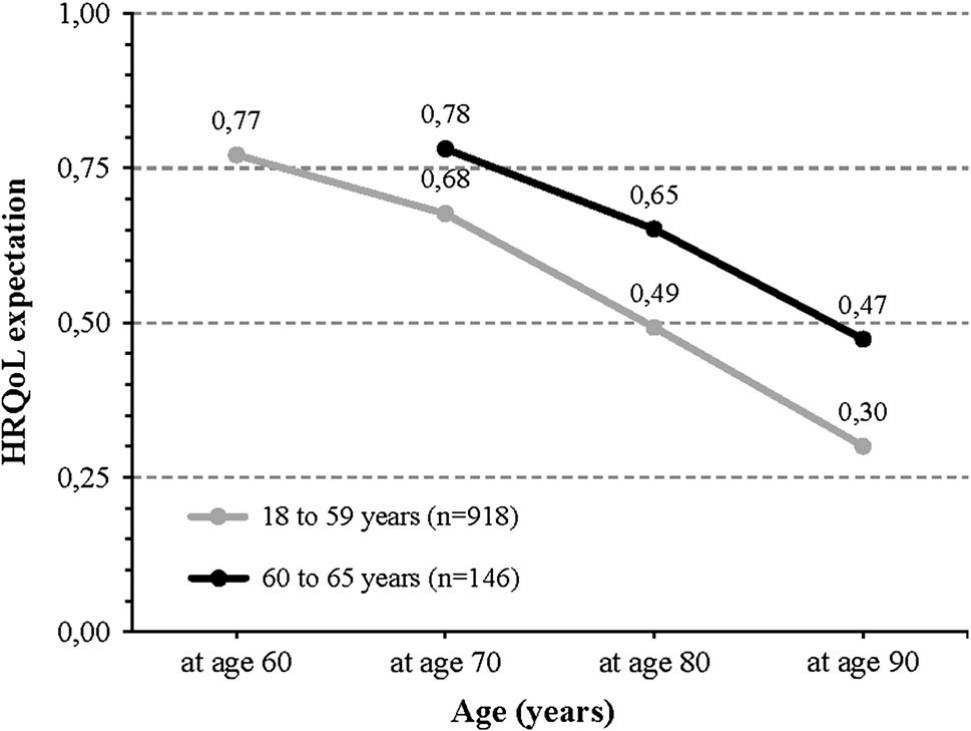
Health-related quality of life (HRQoL) expectations at age 60, 70, 80 and 90 years old, by age group (*n* = 1064)

Figure 5 presents the future health-related quality of life expectations for two age groups, 18–59 and 60–65. As for life expectancy, values were significantly higher for the older group. Interestingly, the initial (i.e., first) reported score was fairly similar for both age groups. The gap between the scores of both groups increased at advanced target ages, from 0.105 to 0.173 at the ages 70 and 90, respectively.

Interestingly, 1.6 % of the respondents indicated the same expected health profiles for all target ages, while an additional 0.6 % of the respondents indicated the same profiles for the ages of 70, 80 and 90. These respondents apparently did not expect their health to deteriorate over time. In addition, 8.4 % of the respondents gave at least one score at a certain target age that was higher than the score at a lower target age.

Respondents were presented with all future health-related quality of life questions despite their subjective life expectancy. Similarly to Brouwer and Van Exel [24] and Péntek et al. [25], further analysis revealed significantly lower scores at the target ages 60–90 for respondents that did not expect to live up to these given ages compared to those who did expect to be alive at these ages. The average scores for the first group and the latter group at ages 60, 70, 80 and 90 were respectively: 0.34 vs 0.79 (Mann–Whitney, *p* < 0.001, non-survivor group *n* = 27), 0.25 vs 0.73 (Mann–Whitney, *p* < 0.001, non-survivor group *n* = 87), 0.30 vs 0.63 (Mann–Whitney, *p* < 0.001, non-survivor group *n* = 377) and 0.26 vs 0.58 (Mann–Whitney, *p* < 0.001, non-survivor group *n* = 852).

### Subjective expectations of remaining number of QALYs from 65 onwards

**Fig. 6:**
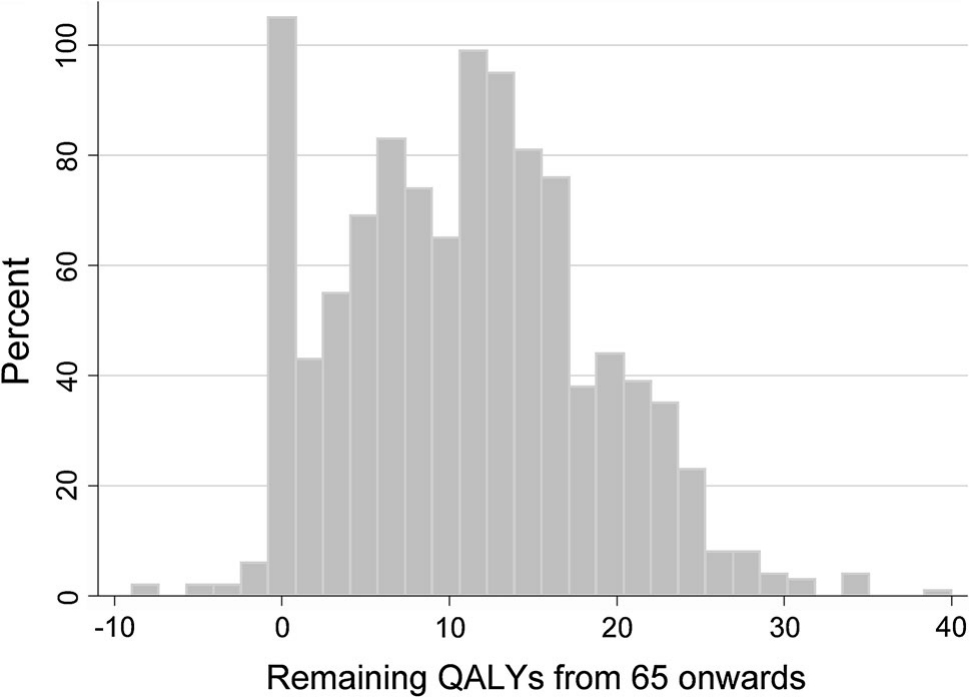
Distribution of self-estimated amount of QALYs from age 65 onwards until expected death (*n* = 1064)

We estimated the number of subjective expected remaining QALYs after age 65 using the information above regarding subjective life expectations and those on future health-related quality of life. Total amount of expected QALYs from 65 onwards to expected death ranged from −9.0 to 40.0 QALYs and mean QALY expectation was 11.0 (SD = 7.4). The distribution of QALY expectations is presented in Fig. 6.

Excluding the lowest and highest 1 % of QALY expectations resulted in QALYs varying between −0.9 and 30.0. Out of the respondents, 3.1 % reported negative QALY expectations, of which two respondents reported expectations lower than −6.0. Such extreme negative QALY expectations can be explained by the fact that these two respondents had already relatively low present self-perceived health status (−0.1 and 0.2), but nonetheless expected to live up to respectively 100 and 92. A longer period of time with such low QALYs scores cumulates to a large negative total of remaining QALYs. Of the respondents, 3.0 % expected to have more than 25 QALYs after the age of 65. All these respondents expected to reach at least 90 years (mean life expectancy of this group is 103 years) in generally good health. One respondent reported a QALY expectation of 40. This respondent reported a life expectancy of 120.

The highest peak was around 0 remaining QALYs. Out of the respondents, 6.4 % self-estimated exactly 0 remaining QALYs after 65. The explanation for this is that these respondents reported life expectancies of 65 or lower. Considering the fact that we did not assign QALYs for the year of expected death, by definition, their total amount of expected future QALYs after 65 amounted to 0.

As expected based on the results above, the mean subjective QALY expectation for the age group 18–59 was 10.5 and significantly lower than the mean expectation of 14.2 of the group 60–65 [*t*(1062) = −5.6353, *p* < 0.001]. Again, no significant variation was found within the age group 18–59.

A small majority of the respondents (56.1 %) preferred a shorter life in perfect health over a longer life in a less then perfect health state. These respondents had significantly lower mean QALY expectations compared to others, respectively: 10.3 vs 11.9 [*t*(1062) = −3.6577, *p* < 0.001].

### Expectations regarding ageing (ERA)

Analysis of the correlation between our future remaining QALY measure and the 12-item ERA resulted in *r* = 0.25, which was significant at the *p* < 0.001 level. The three 4-item subscales correlated in the same direction as the 12-item version of the ERA scale: *r* = 0.20, *r* = 0.20 and *r* = 0.19 (*p* < 0.001 for all correlations) for the expectations regarding physical health scale, mental health scale and cognitive function scale, respectively.

### Multivariate analyses

Table 2 presents the results of the multiple regression analysis with expected remaining QALYs from 65 onwards as dependent variable. We started with a block of background characteristics. Successively, we then added the objective health indicators, two dummy variables representing next of kin’s age of death and finally our lifestyle index.

**Table 2:** Multivariate analysis of remaining QALYs from age 65 to expected death

Variables	Model 1	Model 2	Model 3	Model 4
Male	0.64 (0.468)	0.44 (0.445)	0.63 (0.424)	0.76 (0.420)*
Age dummy	4.82 (0.839)***	4.04 (0.774)***	3.63 (0.761)***	3.45(0.742)***
Age	−0.03 (0.022)	0.03 (0.021)	0.02 (0.020)	0.01 (0.020)
Low education	−1.08 (0.588)*	−1.41 (0.551)**	−1.26 (0.521)**	−1.08 (0.512)**
High education	0.30 (0.541)	0.40 (0.512)	0.39 (0.483)	0.30 (0.477)
Low income	−0.23 (0.581)	0.19 (0.542)	0.13 (0.523)	0.12 (0.517)
High income	0.89 (0.559)	0.66 (0.532)	0.36 (0.493)	0.30 (0.487)
Married	0.58 (0.555)	0.48 (0.521)	0.62 (0.505)	0.39 (0.502)
Have children	0.79 (0.547)	0.43 (0.516)	0.55 (0.498)	0.77 (0.492)
(Self-)employed	1.55 (0.484)***	0.49 (0.465)	0.49 (0.443)	0.45 (0.438)
Chronic disease	–	−2.34 (0.547)***	−2.25 (0.510)***	−2.23 (0.499)***
Disorder	–	−4.02 (0.559)***	−3.73 (0.540)***	−3.66 (0.526)***
Obese	–	−0.20 (0.586)	−0.02 (0.562)	0.08 (0.555)
Next of kin’s age of death low	–	–	−2.93 (0.500)***	−2.71 (0.493)***
Next of kin’s age of death high	–	–	3.44 (0.511)***	3.30 (0.508)***
Lifestyle index	–	–	–	−1.12 (0.210)***
Constant	9.85 (0.966)***	10.01 (0.927)***	9.76 (0.936)***	11.99 (1.021)***
Observations	1064	1064	1064	1064
*R* ^2^	0.07	0.17	0.25	0.27
Adj. *R* ^2^	0.06	0.16	0.24	0.26

Unstandardized coefficients. Robust standard errors in parentheses

*** *p* < 0.01, ** *p* < 0.05, * *p* < 0.10

The fourth, final model explained 27 % of the variance in our outcome variable. In this final model, the age dummy, reflecting the difference between the two age groups 18–59 and 60–65, having a chronic disease and/or disorder, the age of death of next of kin and the lifestyle index were most importantly associated with expectations regarding future QALYs. Less healthy respondents expected to have fewer QALYs from 65 onwards. The same accounts for respondents with an unhealthy lifestyle, a low education and respondents whose next of kin generally died younger. When family members became older, respondents reported higher QALY expectations. For the lifestyle index, each additional type of risky behaviour (i.e. smoking, drinking excessively, etc.) decreased the total amount of future QALYs with 1.12 QALYs. It becomes clear from the beta weights (not shown here) that having a disorder had the strongest effect on the outcome variable. Interestingly, being obese was not a significant explanatory variable for the amount of expected QALYs while being employed was only significant in the first, most restricted model.

We repeated the fourth model of the regression analysis, but replacing the lifestyle index with the individual behavioural risks. Furthermore, we performed the regression analysis for men and women separately. The results are presented in Table 3.

**Table 3:** Multivariate analysis of remaining QALYs from age 65 to expected death: risk factors instead of lifestyle index and distinction male/female

Variables	Model 1	Female	Male
Male	0.76 (0.429)*	–	–
Age dummy	3.40 (0.745)***	1.76 (1.112)	4.65 (1.068)***
Age	0.01 (0.020)	0.04 (0.027)	−0.03 (0.032)
Low education	−1.07 (0.518)**	−0.77 (0.634)	−1.34 (0.860)
High education	0.22 (0.481)	0.51 (0.707)	−0.01 (0.681)
Low income	0.16 (0.521)	−0.23 (0.653)	0.73 (0.889)
High income	0.27 (0.490)	−0.12 (0.759)	0.39 (0.659)
Married	0.41 (0.505)	0.51 (0.724)	0.18 (0.780)
Have children	0.80 (0.491)	0.34 (0.634)	1.34 (0.807)*
(Self-)employed	0.48 (0.441)	1.05 (0.577)*	0.00 (0.698)
Chronic disease	−2.25 (0.502)***	−2.49 (0.710)***	−2.15 (0.720)***
Disorder	−3.64 (0.529)***	−3.46 (0.727)***	−3.85 (0.774)***
Obese	0.01 (0.561)	−0.94 (0.699)	1.17 (0.912)
Next of kin’s age of death low	−2.69 (0.495)***	−3.28 (0.656)***	−1.98 (0.744)***
Next of kin’s age of death high	3.31 (0.511)***	3.17 (0.714)***	3.69 (0.736)***
Smoking	−1.30 (0.450)***	−0.89 (0.605)	−1.56 (0.661)**
No alcohol	−0.09 (0.453)	−0.25 (0.575)	0.13 (0.747)
Excessive alcohol	−0.49 (0.624)	−2.87 (0.931)***	0.59 (0.826)
Physically inactive	−0.78 (0.411)*	0.10 (0.556)	−1.75 (0.623)***
Unhealthy diet	−1.52 (0.421)***	−1.80 (0.558)***	−1.39 (0.631)**
Constant	12.15 (1.014)***	11.08 (1.412)***	14.01 (1.502)***
Observations	1064	531	533
*R* ^2^	0.28	0.29	0.28
Adj. *R* ^2^	0.26	0.27	0.26

Unstandardized coefficients. Robust standard errors in parentheses

*** *p* < 0.01, ** *p* < 0.05, * *p* < 0.10

The regression model in which the lifestyle index was replaced performed similarly in terms of adjusted *R*^2^ to the final model from the regression analysis that included the index. An unhealthy eating habit and smoking were the strongest health behavioural explanatory variables in this model. On average, these variables may be relatively strongly associated in people’s perception with morbidity and mortality, therefore. Both dummies regarding alcohol consumption did not have a significant effect. As can been seen in Table 3, there were some striking differences between men and women regarding the explanatory variables. First, the age dummy had a much stronger effect on expected future QALYs for men than for women. Second, as in the first model shown in Table 3, the alcohol variables were not significant for men. However, excessive alcohol consumption was a significant explanatory variable for the expected future QALYs for women. Finally, the effect of physical inactivity on expectations regarding remaining QALYs only held for men in the separate analyses. Overall, both gender models performed very similarly in terms of explained variance.

### Sensitivity analyses

The final analyses were done to test our findings incorporating some adjustments. First, recall that we only used a QALY score of 0 at time of death for respondents who expected to live beyond 90, since we did not have any expected quality of life score beyond that age. We reran our analysis using a QALY score of 0 for all respondents at the expected age of death. This resulted in a lower mean of remaining future QALYs: 9.5 (SD = 7.2). We repeated the fourth model regression analysis from Table 2 using this estimation. This regression model explained less variance than our original model (*R*^2^ = 0.25 vs *R*^2^ = 0.27) and, furthermore, the significant explanatory variables were less strong in this model than the results shown in Table 2. Replacing the QALY score at time of death with the QALY score at target age 90, instead of a score of 0 for those respondents who expected to live beyond 90 (*n* = 129), slightly increased the mean expected QALYs from 65 onwards to 11.4 QALYs (SD = 8.2). Since the impact of this adjustment seems limited, we did not use this estimate in any further analyses.

Second, our results showed that a few outliers were present both at the minimum and maximum endpoints. A 1 % trimmed mean excluding these outliers resulted in a mean future QALY score of 11.0 (SD = 7.2), ranging from −2.9 to 31.6. We repeated our main regression analysis and this resulted only in minimally lower robust standard errors compared to our original regression analysis from Table 2.

Third, in our analyses we integrated age simultaneously as a continuous variable and as a dummy variable differentiating between age groups 18–59 and 60–65. We tested for several variants of age, e.g., introducing age only as a continuous variable and only as a dummy variable in the regression. The regression model with only age as a continuous variable, which was significant (*p* < 0.001), performed slightly worse in terms of model performance (*R*^2^ = 0.26). No differences were observed for our most important explanatory variables (except for the age weight itself).

Fourth, we used several alternatives to our lifestyle index. The (beta) coefficient of the lifestyle index (as well as the other results) did not alter when we used the original 0–4 score in which the two final categories were not combined or when we applied an index in which the 0–3 score was squared. When we used dummy variables instead of a continuous score of 0–3, i.e., a dummy for score 1 (one lifestyle risk), score 2 (two lifestyle risks) and score 3 (three or four lifestyle risks), we found coefficients of −0.83 (0.573, n.s.), −1.83 (0.571, *p* = 0.001) and −3.56 (0.703, *p* < 0.001), respectively.

## Discussion

In this study, we have presented subjective expectations regarding the amount of QALYs left from 65 onwards until death in a representative Dutch sample of 18- to 65-year-olds in terms of age, gender and level of education. In contrast and addition to previous studies, we have combined expectations regarding length of life and future health-related quality of life into one single measure of healthy life expectation and investigated its relation to a relevant set of background, health and lifestyle variables.

The average amount of subjective expected QALYs from 65 onwards was 11 QALYs and ranged from −9 to 40 QALYs. The final multivariate model from Table 2 explained 27 % of the variance in the amount of future expected QALYs. Lifestyle importantly explained variance in the amount of expected QALYs from 65 onwards. An unhealthier lifestyle was related to lower QALY expectations. Replacing the lifestyle index with the risky behaviours separately—see the first model from Table 3—showed that only individuals who smoke or have poor nutritional habits expect fewer QALYs from 65 onwards. Interestingly, excessive alcohol consumption and physical inactivity did not lower respondents’ subjective QALY expectation. However, interesting gender differences may exist (Table 3). Female heavy drinkers reported significantly lower expectations, but this did not hold for men. Smoking and physical inactivity, however, were only associated with a lower amount of expected QALYs for male respondents. It should be noted, however, that the relation of excessive alcohol consumption and smoking and QALY expectations showed a somewhat similar pattern for both genders (except for their statistical significance). In other words, both risky behaviours were associated with lower expectations for both men and women, but with a slightly different magnitude. Moreover, the group of female excessive alcohol consumers was rather small (*n* = 40), which may have influenced our results. The impact of an unhealthy diet on the number of expected QALYs was similar for both men and women. The association between the expected future QALYs and lifestyle and possible differences between men and women in this respect, especially regarding alcohol consumption, warrant further investigation.

Another important point here is that the causality of the relation between QALY expectations and lifestyle may work in both directions. On the one hand, individuals with an unhealthy lifestyle may incorporate the adverse consequences of their behaviour into their QALY expectations and adjust their expectations downwards. On the other hand, individuals with low QALY expectations may adopt an unhealthy lifestyle since they may believe that unhealthy habits do not matter that much for them (given low expectations) or may feel unable to influence their expectations regarding length and future health-related quality of life. This may be related to the findings of Sarkisian et al. [3] regarding seeking medical treatment. It would be interesting to study this circular relationship in more detail.

Our multivariate regression analysis further showed that respondents with a severe disorder (now or in the past) or chronic disease expected fewer QALYs in the future compared to healthy respondents. Interestingly, being obese did not explain any variance in our outcome variable. Although respondents with a disorder (now or in the past) and/or chronic disease had significantly higher BMI scores, excluding obesity or, alternatively, the variables regarding having a disorder or chronic disease, did not alter any of the relevant coefficients. Finally, the average age of death of next of kin predicted our outcome variable as well, in the expected direction, as was found before [24, 25].

### Limitations

A few limitations of our study should be taken into account when interpreting our results. First, we excluded a considerable proportion (i.e. 12.8 %) of initial respondents, largely based on supposed speeding through the online questionnaire. Consequently, the final sample available for analysis was no longer completely representative of the Dutch population, with younger and male respondents slightly underrepresented. However, since mean scores on our main outcome measure did not differ significantly between included and excluded respondents, we believe that elimination of respondents did not introduce a disturbing selection bias, and therefore does not greatly affect the generalizability of our results.

Second, the EQ-5D is a validated instrument and widely applied as a health outcome measure. However, its use for eliciting expectations regarding health-related future quality of life is less common. We slightly adjusted the wording of the EQ-5D questions to make the instrument suitable for obtaining health expectations, analogous to the format used by Brouwer and Van Exel [24] and Péntek et al. [25]. These authors concluded that individuals seem to answer the questions as intended, since the scores for expected and actual health at age 60 were similar. The correlation of our outcome variable and the ERA provides some further validation for our application of the EQ-5D. Obviously, further validation is required and exploring other methods for obtaining expectations of future health is encouraged.

Third, the design of our survey and the questions posed to the respondents may have influenced our results. For example, in the expectation section of our questionnaire, respondents were first asked to indicate their subjective life expectancy. Then we administered the EQ-5D to elicit expectations regarding future health-related quality of life. It is unclear whether this sequence influenced respondents’ answers. Moreover, respondents answered the future health questions successively for the target ages 60, 70, 80 and 90 years old. This may induce respondents to indicate a decline in health with age.

Fourth, respondents answered all questions regarding expectations of future health despite their subjective life expectancy. As Brouwer and Van Exel [24] noted in this context, “…one may expect that health-related quality of life expectations for ages at which one does not believe to be alive anymore are irrelevant and perhaps unrealistically low, because respondents try to indicate their expectation of longevity in the indicated health profile.” Indeed, we found significantly lower quality of life expectations for ‘non-survivors’ vs. ‘survivors’, which raises the question of the validity of answers to questions regarding future health-related quality of life beyond the expected age of death.

Another point is that more explanatory variables could have been included in this study. For instance, it could have been interesting to investigate the associations between future health expectations and choices related to saving and insurance coverage. These are interesting options for future research.

A final limitation is that we did not explicitly ask about the expected quality of life close to the time of death. We therefore imputed these scores. The sensitivity analysis showed that using a QALY score of 0 did alter our findings somewhat. This may be investigated in more detail in future research.

### Age

The role of age in our analyses should be interpreted with some caution. We found that respondents aged 60–65 reported significantly higher QALY expectations than younger respondents (see the coefficient of the age dummy in Table 2). For respondents in this older age group, we calculated the amount of expected QALYs for the time frame 65–70 differently, i.e., we used their current self-reported health state instead of their quality of life expectation at target age 60 (see Fig. 3). Nonetheless, we observed higher QALY expectations for the 60- to 65-year-old respondents also for the age periods of 70–80, 80–90 and 90-death, as well as higher expected quality of life scores at 70, 80 and 90 (Fig. 5) and a higher subjective life expectancy. Therefore, our computation method does not explain the higher expectations of the older age group. A possible explanation for the fact that we found higher expectations for the age group 60–65 than for the other respondents is that achieving a certain age (in a certain health state) may increase expectations. Indeed, the expectations that young and middle-aged adults have about ageing may differ importantly from those of older adults who have more experience with ageing. The negative images associated with ageing such as illness, memory loss, dependence on others and loneliness may differ between age groups as well. Moreover, younger individuals may draw the line between young and old at a lower age than older people do [33].

Interestingly, more than half of the respondents in the age group 60–65 (*n* = 146) were retired. This group of ‘early retirees’ reported a better mean current health state and a significantly higher amount of expected QALYs compared to the other respondents in our sample, 15.8 QALYs vs 10.6 QALYs, respectively. Retirees’ QALY expectation was also significantly higher than the other respondents within the age group 60–65. This effect on the amount of expected QALYs only held for men when conducting our multivariate regression analysis for men and women separately, which may be explained by the fact that 81 % of the retirees were male.

### Subjective life expectancy and future health-related quality of life

Explanatory variables may be associated with either subjective life expectancy or future health-related quality of life, or with both. Brouwer and Van Exel [24] mainly found significant associations between age, health status and perception of own lifestyle compared to others and both types of expectations, whereas the average age of death of family members only related to subjective life expectancy. Péntek et al. [25] found similar results for expected health (but kin’s age of death was also significantly related to expected health), whereas all included explanatory variables were significantly related to subjective life expectancy (also due to their large sample size).

Although our study methods and sample in some respects differed from the methods used in these studies, our analyses for our composite outcome indicator of expectations leads to similar conclusions.^2^ We conducted separate regression analyses similar to those in Table 3 using subjective life expectancy and expected health as dependent variables. First, we found that having children and smoking became especially relevant in explaining the variance in subjective life expectancy. Second, having a chronic disease was only significantly related to expectations regarding future health-related quality of life. Drinking behaviour (both abstaining and drinking excessively) and physical inactivity were slightly negatively associated with future health at age 65, while an unhealthy diet mainly played a role regarding future health at older ages. Third, age of death of relatives was related to expectations regarding both length and quality of life. These results altered somewhat when the analyses were conducted for men and women separately. These additional analyses indicate that individuals relate different consequences in terms of life expectancy and future health-related quality of life to different behaviours. Moreover, apparently men and women perceive some risks differently. These are important implications for designing health promotion strategies targeted at specific unhealthy behaviours and groups.

## Conclusion

In conclusion, we combined two concepts of expectations into one composite indicator of the expected amount of QALYs from the age of 65 onwards until death. With this, we extended the concept of subjective life expectancy by correcting expected longevity for the expected quality of life during these years. As such, it provides more information than subjective life expectancy alone and therefore may prove more valuable for understanding people’s perceptions regarding ageing and, consequently, demand for health services and long-term care needs. It may also provide important information on the perceived impact of health behaviour on expectations (and vice versa), which could be relevant for health policy strategies aimed at improving lifestyles. More insight into individuals’ subjective expectations remains warranted.

## Footnotes

1 In this paper, when we refer to QALYs or expected QALYs, we mean subjective QALY expectations.

2 The average subjective life expectancy in our study was 1.5 years higher than the mean found by Péntek et al. [25], and 2 years lower than the mean found by Brouwer and Van Exel [24]. Furthermore, our results showed a much more gradual decline in health with age than the expected sharp decline found in these previous studies, i.e. from around 0.8 at age 60 to around 0.06 at age 90. However, comparisons should be interpreted with caution due to the different study samples (for example, mean age was around 8 years higher in the present study) and study design.
